# Human Papillomaviruses Genotype Distribution and Replacement by Year from 2014 to 2020 in Beijing, China

**DOI:** 10.1089/whr.2023.0153

**Published:** 2024-05-17

**Authors:** Xu Ma, Xiaoli Zeng, Hui Yuan

**Affiliations:** Clinical Laboratory, Beijing Anzhen Hospital Affiliated of Capital Medical University, Beijing Institute of Heart, Lung and Blood Vessel Diseases, Beijing, P.R. China.

**Keywords:** genotype, human papillomavirus, prevalence, replacement, women

## Abstract

**Background:**

Cervical cancer is one of the most common gynecological malignancies worldwide. The incidence of cervical cancer ranks second for female malignancies in China. However, human papillomavirus (HPV) prevalence and genotype replacement for a long time in Beijing were not yet evaluated.

**Methods:**

Women patients who visited clinical departments, especially the gynecology department, were included in this study from 2014 to 2020. They suffered from different kinds of cervical abnormalities even cervical cancer. Thirteen types of HPV were detected in cervical samples by using multiple fluorescence polymerase chain reactions. All patients were divided into four groups according to age (15–30, 31–45, 46–60, and >60 years old).

**Results:**

The study included data from four certain years (2014, 2016, 2018, and 2020). Overall HPV prevalence was 18.0%, 16.7%, 21.9%, and 19.1%, respectively. Of the 13 genotypes, the top one infection type was HPV52, followed by HPV58 and HPV16 or HPV16 and HPV58 in different years. HPV56 prevalence raised from 2018, which replaced HPV39 into the top five list. Only HPV prevalence of 46–60 years age group declined, mainly resulting from the reduced prevalence of HPV39 and HPV58 (*p* < 0.001 and *p* = 0.020). The proportions of HPV dual-infection across the four years varied significantly in statistics (*p* = 0.001), whereas the most common dual-infection HPV39/68 disappeared after 2018.

**Conclusion:**

The prevalence of two HPV genotypes (HPV39 and HPV58) and dual-infection HPV39/68 showed a declining trend, especially in the 46–60 years age group. The trends need to be observed continuously.

## Introduction

Cervical cancer is the fourth most frequent cancer in women worldwide, representing nearly 8% of all female cancer deaths every year.^1^ Epidemiological studies report that approximately 95% of cases of cervical cancer are caused by human papillomavirus (HPV).^2,3^ In China, 130,000 new cases of cervical cancer are diagnosed annually, accounting for 28% of the world’s total.^4^ Detailed understanding of high-risk HPV type distribution can help inform vaccine research, development, and allocation.

HPV is a sexually transmitted virus and more than 200 HPVs have been identified and completely sequenced.^5^ The virus is classified into high-risk and low-risk types based on oncogenic potential. In a large proportion of cases, the infections were eliminated without symptoms, but in some women, the infections with high-risk HPV became persistent and caused precancerous lesions and cancers.^6^ In 2020, an estimated 604,237 women were diagnosed with cervical cancer globally, representing 6.5% of all female cancers.

To date, the Chinese government has approved three imported vaccines against HPV infection. In July 2016, the bivalent vaccine Cervarix against HPV16 and 18 was approved by China first. Then the Gardasil-4 vaccine against HPV 6/11/16/18 was approved in May 2017. One year later, the Gardasil-9 vaccine, which can prevent the infection of HPV6/11/16/18/31/33/45/52/58, was approved to use by hospitals in April 2018. Studies earlier showed that in many regions worldwide, HPV16 and 18 were the cause of approximately 70% of all cervical cancers, as well as a subset of anogenital and oropharyngeal cancers.^7^ However, a study carried out by West China Hospital screening cervical cancer in older than 20 years women between January 2015 and December 2016 found that only 25.9% of the high-grade squamous intraepithelial lesion (HSIL) cases were HPV16/18 positive, while up to 56.8% HSIL cases were positive of HPV31/33/45/52/58.^8^

As introduced earlier, 9-valent Gardasil-9 vaccine covers seven high-risk HPV types (HPV16/18/31/33/45/52/58), besides two low-risk HPV types (HPV6/11). In fact, high-risk HPVs consist of 15 types (HPV16/18/31/33/35/39/45/51/52/56/58/59/66/68/82) or even more. The prevalent types vary largely in different areas. A large meta-analysis of 2010 reported that the five most common types in five continents (Europe, Africa, Latin America and Caribbean, Northern America, and Asia) were HPV16 (3.2%), HPV18 (1.4%), HPV52 (0.9%), HPV31 (0.8%), and HPV58 (0.7%) in the pre-HPV vaccination era. Although HPV33/45 were not the most prevalent types worldwide, they were very common in Latin America or Africa.^9^

As mentioned before, HPV vaccines have been approved successively by the Chinese government since July 2016, protecting girls and women aged 9–45 years from HPV infection. However, data on HPV genotype distribution among women during a long time especially before and after HPV vaccination is scarce. Therefore, we performed this study in which HPV genotype distribution was described and analyzed in four specific years during 2014–2020 (2014, 2016, 2018, and 2020), with aiming to improve vaccination and HPV-based cervical screening strategies.

## Materials and Methods

### Study population

The study was approved by the Ethics Committee of Beijing Anzhen Hospital, China, and was conducted according to the principles of the Declaration of Helsinki. We collected female patients across 7 years, including the years 2014, 2016, 2018, and 2020. Female patients who visited gynecology department (not including the obstetrics department), dermatology department, and other departments in Beijing Anzhen Hospital and carried out high-risk HPV detection were enrolled in this study. The cases of the Health Management Center were excluded. Female patients who were below the age of 15 years were also excluded from the study.

### HPV–DNA extraction and genotyping

Cervical cells were obtained from the cervical canal using one plastic brush. The brush was placed into a 3-mL vial of preservation solution (Liferiver, Shanghai, China) for subsequent HPV–DNA testing. Thirteen high-risk HPV genotypes (HPV16/18/31/33/35/39/45/51/52/56/58/59/68) and one human house-keeping gene as internal control were simultaneously amplified in four 0.2 mL polymerase chain reaction (PCR) reaction tubes. Internal control positive amplification indicated sampling acceptable.

The samples for HPV–DNA tests were stored at room temperature for no more than 1 day, or at 2°C–8°C for no more than 7 days. Pyrolysis method was applied to extract total DNA in samples until July 2016. Magnetic beads method was applied to extract total DNA on Autrax192 automatic nucleic acid workstation (Liferiver, Shanghai, China) from August 2016. The method of multiplex fluorescence PCR (Liferiver, Shanghai, China) was used for high-risk HPV genotyping.

### Statistical analysis

Data of 4 years (2014, 2016, 2018, and 2020) was analyzed by SPSS 22.0 for Windows. Chi-squared (χ^2^) Testor Fisher’s exact test was adopted to examine the significance of frequency distributions. *p* < 0.05 was considered statistically significant. Prevalence of overall HPV infection, each type and multiple type of infection were analyzed, respectively. Top five genotypes were analyzed in detail, as well as dual-genotype infection. To explore the difference in infection rate by age, the total population was divided into four groups (15–30, 31–45, 46–60, and >60 years age groups). Each type of prevalence and overall prevalence in each age group were all analyzed and compared. Because of the highest risk of HPV16/18, the combined prevalence of these two types was showed particularly.

## Results

### Prevalence change of high-risk HPV infection by year

The case number retrieved in 2014, 2016, 2018, and 2020 was 6939, 5992, 6936, and 4544, respectively. Patients who were infected one or more high-risk HPV were classified as positive cases. The overall prevalence rate was at 18.0%, 16.7%, 21.9%, and 19.1%, respectively (χ^2^ = 63.86, *p* < 0.001). The prevalence of each genotype is shown in Table 1. In 2014, the prevalence of single type ranged from 0.3% to 4.8%. In 2016, it ranged from 0.3% to 3.8%, with nine genotypes prevalence lower than in 2014. After 2016, the prevalence increased even beyond the prevalence of 2014, ranging from 0.6% to 5.1% in 2018, and ranging from 0.3% to 5.0% in 2020. But the total prevalence of 13 genotypes in 2020 was not significantly different, compared with the total prevalence in 2014 (19.1% *vs.* 18.0%, *p* = 0.136).

**TABLE 1.** Overall Prevalence and Each Genotype Prevalence of High-Risk HPV in Studied Years

HPV type	2014	2016	2018	2020	Four years	2014 *vs.* 2020	Trend^a^
	*n* = 6939	*n* = 5992	*n* = 6936	*n* = 4544			
16	203 (2.9)	182 (3.0)	300 (4.3)	149 (3.3)	<0.001	0.282	
18	108 (1.6)	70 (1.2)	102 (1.5)	72 (1.6)	0.213	0.906	
31	62 (0.9)	50 (0.8)	114 (1.6)	83 (1.8)	<0.001	<0.001	↑
56	93 (1.3)	98 (1.6)	169 (2.4)	105 (2.3)	<0.001	<0.001	↑
45	23 (0.3)	20 (0.3)	41 (0.6)	15 (0.3)	0.041	0.990	
35	45 (0.6)	40 (0.7)	61 (0.9)	44 (1.0)	0.138	0.056	
59	100 (1.4)	76 (1.3)	115 (1.7)	65 (1.4)	0.325	0.963	
39	176 (2.5)	107 (1.8)	157 (2.3)	80 (1.8)	0.006	0.006	↓
51	160 (2.3)	116 (1.9)	187 (2.7)	115 (2.5)	0.033	0.441	
58	230 (3.3)	184 (3.1)	261 (3.8)	146 (3.2)	0.148	0.765	
52	335 (4.8)	226 (3.8)	357 (5.1)	226 (5.0)	0.001	0.723	
33	53 (0.8)	33 (0.6)	60 (0.9)	36 (0.8)	0.206	0.865	
68	85 (1.2)	53 (0.9)	131 (1.9)	56 (1.2)	<0.001	0.972	
Total	1252 (18.0)	998 (16.7)	1520 (21.9)	870 (19.1)	<0.001	0.136	

^a^ “↑” or “↓” indicates that certain prevalence changed statistically significantly among those 4 years as well as between 2014 and 2020. HPV, human papillomavirus.

Among the studied four years, the prevalence of five genotypes (HPV18/35/59/58/33) showed no statistically significant variations (all *p* > 0.05, Table 1), while the prevalence of the other eight genotypes changed statistically significantly (*p* <0.05 or <0.001). However, if the prevalence of 2020 was compared with 2014, only the prevalence of three HPV types (HPV31, 56, and 39) showed statistically significant variations (*p* < 0.001, *p* < 0.001, and *p* = 0.006, respectively, Table 1).

### Prevalence change of high-risk HPV infection of different numbers by year

**FIG. 1.**
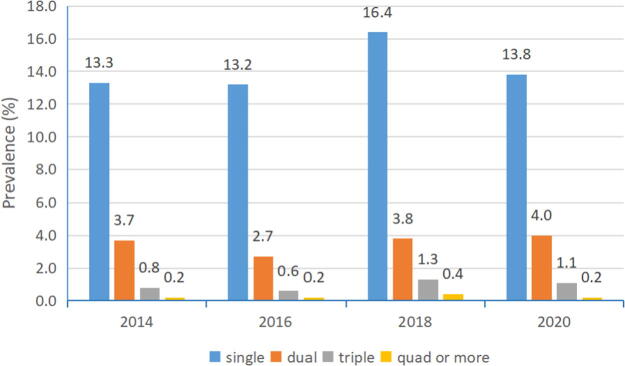
The prevalence of single and more types of high-risk HPV infection in studied years. HPV, human papillomavirus.

Single-genotype infection accounted for the major proportion of total infection (Fig. 1). Dual infection was the second major infection type. The prevalence of dual infection was at 3.7%, 2.7%, 3.8%, and 4.0% during 2014–2020, respectively (Table 2). It was shown that the dual infection prevalence among those 4 years was statistically significant (χ^2^ = 16.08, *p* = 0.001). The triple genotype infection might show an increase trend after 2016 (χ^2^ = 20.59, *p* < 0.001).

**TABLE 2.** Prevalence of High-Risk HPV Infection of Different Number

No. of infected types	2014	2016	2018	2020	χ^2^	*p*
	*n* = 6939	*n* = 5992	*n* = 6936	*n* = 4544		
1	925 (13.3)	793 (13.2)	1140 (16.4)	626 (13.8)	37.76	<0.001
2	254 (3.7)	163 (2.7)	265 (3.8)	180 (4.0)	16.08	0.001
3	56 (0.8)	33 (0.6)	87 (1.3)	52 (1.1)	20.59	<0.001
>3	17 (0.2)	9 (0.2)	28 (0.4)	12 (0.2)	/	/
Total	1252 (18.0)	998 (16.7)	1520 (21.9)	870 (19.1)	63.86	<0.001

HPV, human papillomavirus.

### High-risk HPV prevalence in different age groups by year

**FIG. 2.**
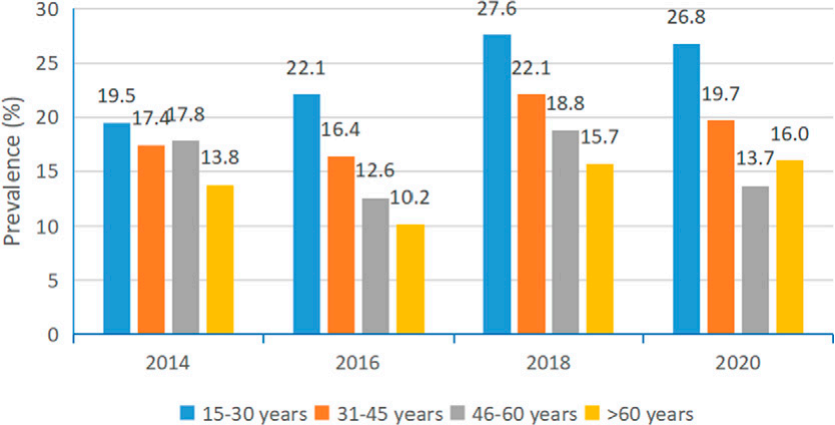
Age-specific overall HPV prevalence in studied years. HPV, human papillomavirus.

Figure 2 and Tables 3–6 presented age-specific HPV prevalence by year. In any year, a peak of HPV infection was found in younger age group (15–30 years). As age increased, the prevalence gradually declined except for the >60 years age group of 2020. The prevalence (16.0%) was evidently higher than the prevalence of the 46–60 years age group which was 13.7% (χ^2^ = 89.11, *p* < 0.001, not showed in tables). Although the 9-valent vaccine was on the Chinese market in 2018, the overall prevalence (not significant, *p* = 0.136, Table 1) and the prevalence of our two younger age groups in 2020 was higher than in the year 2014 (*p* < 0.001, Table 3; *p* = 0.001, Table 4; Fig. 2). While the prevalence of the 46–60 age group in 2020 was lower than in the year 2014 (*p* = 0.024, Table 5).

**TABLE 3.** Each Genotype Prevalence of High-Risk HPV in 15–30 Years Age Group

HPV type	2014	2016	2018	2020	Four years	2014 *vs.* 2020	Trend^a^
	*n* = 2606	*n* = 1754	*n* = 1773	*n* = 1037			
16	89 (3.4)	73 (4.2)	120 (6.8)	51 (4.9)	<0.001	0.033	↑
18	52 (2.0)	35 (2.0)	41 (2.3)	32 (3.1)	0.200	0.048	
31	22 (0.8)	18 (1.0)	38 (2.1)	30 (2.9)	<0.001	<0.001	↑
56	32 (1.2)	42 (2.4)	57 (3.2)	42 (4.1)	<0.001	<0.001	↑
45	6 (0.2)	5 (0.3)	12 (0.7)	2 (0.2)	0.058	0.828	
35	18 (0.7)	9 (0.5)	23 (1.3)	18 (1.7)	0.002	0.004	↑
59	44 (1.7)	33 (1.9)	52 (2.9)	24 (2.3)	0.035	0.208	
39	77 (3.0)	49 (2.8)	55 (3.1)	28 (2.7)	0.920	0.679	
51	73 (2.8)	53 (3.0)	76 (4.3)	46 (4.4)	0.012	0.012	↑
58	97 (3.7)	63 (3.6)	88 (5.0)	52 (5.0)	0.060	0.076	
52	141 (5.4)	88 (5.0)	112 (6.3)	66 (6.4)	0.257	0.262	
33	22 (0.8)	13 (0.7)	24 (1.4)	12 (1.2)	0.225	0.375	
68	34 (1.3)	25 (1.4)	50 (2.8)	14 (1.4)	0.001	0.914	
Total	507 (19.5)	387 (22.1)	490 (27.6)	278 (26.8)	<0.001	<0.001	↑

^a^ “↑” or “↓” indicates that certain prevalence changed statistically significantly among those 4 years as well as between 2014 and 2020. HPV, human papillomavirus.

**TABLE 4.** Each Genotype Prevalence of High-Risk HPV in 31–45 Years Age Group

HPV type	2014	2016	2018	2020	Four years	2014 *vs.* 2020	Trend^a^
	*n* = 2686	*n* = 2329	*n* = 2467	*n* = 1661			
16	74 (2.8)	69 (3.0)	91 (3.7)	60 (3.6)	0.177	0.112	
18	39 (1.5)	22 (0.9)	37 (1.5)	23 (1.4)	0.316	0.856	
31	24 (0.9)	21 (0.9)	46 (1.9)	34 (2.0)	<0.001	0.001	↑
56	36 (1.3)	28 (1.2)	51 (2.1)	36 (2.2)	0.020	0.038	↑
45	9 (0.3)	7 (0.3)	14 (0.6)	9 (0.5)	0.380	0.302	
35	16 (0.6)	14 (0.6)	17 (0.7)	15 (0.9)	0.631	0.242	
59	36 (1.3)	34 (1.5)	42 (1.7)	26 (1.6)	0.751	0.543	
39	62 (2.3)	32 (1.4)	57 (2.3)	33 (2.0)	0.068	0.481	
51	53 (2.0)	39 (1.7)	68 (2.8)	41 (2.5)	0.052	0.275	
58	74 (2.8)	83 (3.6)	80 (3.2)	45 (2.7)	0.289	0.928	
52	129 (4.8)	82 (3.5)	133 (5.4)	88 (5.3)	0.011	0.466	
33	22 (0.8)	15 (0.6)	19 (0.8)	11 (0.7)	0.876	0.563	
68	34 (1.3)	17 (0.7)	37 (1.5)	16 (1.0)	0.067	0.363	
Total	467 (17.4)	382 (16.4)	546 (22.1)	328 (19.7)	<0.001	0.001	↑

^a^ “↑” or “↓” indicates that certain prevalence changed statistically significantly among those 4 years as well as between 2014 and 2020. HPV, human papillomavirus.

**TABLE 5.** Each Genotype Prevalence of High-Risk HPV in 46–60 Years Age Group

HPV type	2014	2016	2018	2020	Four years	2014 *vs.* 2020	Trend^a^
	*n* = 1277	*n* = 1421	*n* = 1982	*n* = 1347			
16	34 (2.7)	31 (2.2)	69 (3.5)	28 (2.1)	0.044	0.325	
18	14 (1.1)	8 (0.6)	18 (0.9)	13 (1.0)	0.484	0.739	
31	14 (1.1)	11 (0.8)	24 (1.2)	17 (1.3)	0.584	0.695	
56	17 (1.3)	17 (1.2)	49 (2.5)	17 (1.3)	0.007	0.876	
45	7 (0.5)	7 (0.5)	12 (0.6)	3 (0.2)	0.450	0.176	
35	9 (0.7)	12 (0.8)	14 (0.7)	7 (0.5)	0.786	0.543	
59	15 (1.2)	8 (0.6)	12 (0.6)	10 (0.7)	0.227	0.255	
39	33 (2.6)	20 (1.4)	35 (1.8)	10 (0.7)	0.002	<0.001	↓
51	27 (2.1)	18 (1.3)	29 (1.5)	17 (1.3)	0.232	0.089	
58	47 (3.7)	26 (1.8)	67 (3.4)	29 (2.2)	0.005	0.020	↓
52	48 (3.8)	45 (3.2)	89 (4.5)	51 (3.8)	0.260	0.971	
33	8 (0.6)	3 (0.2)	11 (0.6)	10 (0.7)	0.247	0.719	
68	17 (1.3)	9 (0.6)	36 (1.8)	16 (1.2)	0.026	0.742	
Total	227 (17.8)	179 (12.6)	372 (18.8)	184 (13.7)	<0.001	0.024	↓

^a^ “↑” or “↓” indicates that certain prevalence changed statistically significantly among those four years as well as between 2014 and 2020. HPV, human papillomavirus.

**TABLE 6.** Each Genotype Prevalence of High-Risk HPV in >60 Years Age Group

HPV type	2014	2016	2018	2020	Four years	2014 *vs.* 2020	Trend^a^
	*n* = 370	*n* = 488	*n* = 714	*n* = 499			
16	6 (1.6)	9 (1.8)	20 (2.8)	10 (2.0)	0.538	0.678	
18	3 (0.8)	5 (1.0)	6 (0.8)	4 (0.8)	0.980	0.988	
31	2 (0.5)	0 (0.0)	6 (0.8)	2 (0.4)	0.225	0.763	
56	8 (2.2)	11 (2.3)	12 (1.7)	10 (2.0)	0.900	0.871	
45	1 (0.3)	1 (0.2)	3 (0.4)	1 (0.2)	0.877	1.000^*^	
35	2 (0.5)	5 (1.0)	7 (1.0)	4 (0.8)	0.866	0.646	
59	5 (1.4)	1 (0.2)	9 (1.3)	5 (1.0)	0.242	0.633	
39	4 (1.1)	6 (1.2)	10 (1.4)	9 (1.8)	0.810	0.386	
51	7 (1.9)	6 (1.2)	14 (2.0)	11 (2.2)	0.696	0.749	
58	12 (3.2)	12 (2.5)	26 (3.6)	20 (4.0)	0.564	0.554	
52	17 (4.6)	11 (2.3)	23 (3.2)	21 (4.2)	0.213	0.783	
33	1 (0.3)	2 (0.4)	6 (0.8)	3 (0.6)	0.636	0.476	
68	0 (0.0)	2 (0.4)	8 (1.1)	10 (2.0)	0.012	0.006	↑
Total	51 (13.8)	50 (10.2)	112 (15.7)	80 (16.0)	0.029	0.228	

^a^ “↑” or “↓” indicates that certain prevalence changed statistically significantly among those four years as well as between 2014 and 2020. HPV, human papillomavirus.

^*^ indicated the *p* value was resulted from Fisher’s exact test rather than chi-square test.

According to Table 3, we could find that six HPV types showed higher prevalence in 2020 than in 2014 among the 15- to 30-year-old women population (all *p* < 0.05). Similarly, two HPV types (HPV31/56) showed higher prevalence among the 31- to 45-year-old women population (both *p* < 0.05, Table 4). However, the prevalence of the other two HPV types became reduced in the 46–60 years age group, including HPV39/58 (both *p* < 0.05, Table 5). Differing from the above three groups, in the >60 years age group most HPV types’ prevalence showed no fluctuation between 2020 and 2014, except one type (HPV68) prevalence increasing (*p* = 0.006, Table 6).

### Top five infection genotypes of high-risk HPV

We listed the top five HPV genotypes for total positive rate in each year (Table 7). HPV52 was the top one with the highest prevalence at 5.1% in 2018 and the lowest at 3.8% in 2016 (*p* = 0.001, see Table 1). HPV58 and HPV16 were the second or the third top genotypes (*p* = 0.148, *p* < 0.001, respectively, see Table 1). HPV39 prevalence fell from the top fourth in 2014 to the five-below in 2020 (*p* = 0.006, see Table 1). HPV56 prevalence increased which appeared in the fifth place in 2018 and 2020 (*p* < 0.001, see Table 1). According to those results presented in Tables 3–6, it could be found that HPV56 was prevalent in the older female group in 2014 and 2016, but it became prevalent in all age groups in 2018 and 2020.

**TABLE 7.** Top Five Infection Genotypes of High-Risk HPV in Studied Years

2014 (*n* = 6939)		2016 (*n* = 5992)		2018 (*n* = 6936)		2020 (*n* = 4544)	
HPV type	Total Prevalence	HPV type	Total prevalence	HPV type	Total prevalence	HPV type	Total prevalence
52	335 (4.8)	52	226 (3.8)	52	357 (5.1)	52	226 (5.0)
58	230 (3.3)	58	184 (3.1)	16	300 (4.3)	16	149 (3.3)
16	203 (2.9)	16	182 (3.0)	58	261 (3.8)	58	146 (3.2)
39	176 (2.5)	51	116 (1.9)	51	187 (2.7)	51	115 (2.5)
51	160 (2.3)	39	107 (1.8)	56	169 (2.4)	56	105 (2.3)
Total combined prevalence	1104 (15.8)	Total combined prevalence	815 (13.6)	Total combined prevalence	1274 (18.3)	Total combined prevalence	741 (16.3)

HPV, human papillomavirus.

### Prevalence change of HPV16/18 overall infection by year

**FIG. 3.**
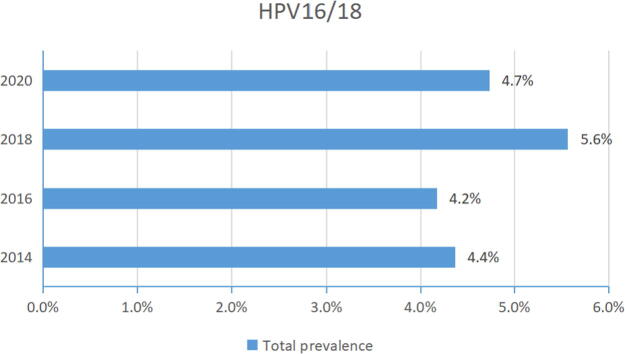
Total prevalence of two high-risk HPV (HPV16 and 18) by year. HPV, human papillomavirus.

HPV16 and HPV18 have been assumed the most risky genotypes. So we analyzed the combined prevalence of the two genotypes, including single and multiple infections. Similar to the overall prevalence of all genotypes (Table 1), HPV16/18 total prevalence showed a wavy line across the seven years (Fig. 3). The prevalence of HPV16/18 in each year was 4.4%, 4.2%, 5.6%, and 4.7%, respectively.

### Top five dual-infection genotypes of high-risk HPV

The first common dual-infection genotypes were HPV39/68 from 2014 until 2018, then HPV52/58 rising to the top one in 2020 and HPV39/68 disappearing from the top five list (Table 8). Except the most common type HPV39/68, other dual-infection genotypes were almost composed of HPV52, HPV58, and HPV16. In addition, HPV51 was another main genotype of HPV dual infection. HPV51/52 was the third common dual-infection in 2014, 2016, and 2020. Along with the increase of HPV56 overall prevalence since 2016 possibly (Table 7), the dual infection of HPV51/56 rose to the fourth in 2020. And a new dual-infection genotype HPV16/33 was prevalent at 3.3%, being the fifth common dual-infection of 2020.

**TABLE 8.** Top Five Dual-Infection Genotypes of High-Risk HPV in Studied Years

2014 (*n* = 254)		2016 (*n* = 163)		2018 (*n* = 265)		2020 (*n* = 180)	
HPV types	Prevalence	HPV types	Prevalence	HPV types	Prevalence	HPV types	Prevalence
39/68	35 (13.8)	39/68	13 (8.0)	39/68	29 (10.9)	52/58	13 (7.2)
52/58	24 (9.4)	16/51	9 (5.5)	16/52	16 (6.0)	16/52	10 (5.6)
51/52	13 (5.1)	51/52	9 (5.5)	52/58	13 (4.9)	51/52	9 (5.0)
16/52	11 (4.3)	39/52	8 (4.9)	16/58	9 (3.4)	51/56	7 (3.9)
52/59	10 (3.9)	52/58	7 (4.3)	39/52	9 (3.4)	16/33	6 (3.3)

HPV, human papillomavirus.

## Discussion

The geographical differences in HPV genotype distribution have been compared in a vast number of areas worldwide as well as in China, while the temporal changes in HPV genotype distribution seemed to be of less attention. In the study, we analyzed HPV genotype distribution yearly during seven years to better interpret changes in HPV genotype distribution which resulted from various factors including vaccination.

According to our results (Table 1), the overall prevalence of all HPV types in Beijing is presented as a wavy line. They were high in 2014 and 2018 and were relatively low in 2016 and 2020. The prevalence differences of eight HPV types among those 4 years were statistically significant (*p* < 0.05). But when the comparisons were conducted between 2014 and 2020, the prevalence differences of 10 genotypes were not statistically significant (*p* > 0.05). Only HPV31/56 prevalence increased (*p* < 0.05) and HPV39 prevalence reduced (*p* < 0.05). An observational study comparing HPV prevalence and type distribution by Enerly et al. showed that vaccine-targeted HPV types (HPV16 or 18) in the vagina of vaccinated girls from the first birth cohort eligible for school-based HPV vaccination in Norway had a lower prevalence (0.4% *vs.* 4.1%, prevalence ratio: 0.10, 95% CI: 0.01–0.98).^10^ However, in our study there was no decrease in infection rates for HPV16 or 18 between 2014 and 2020, perhaps inferring that the three HPV vaccines had not significantly affected the transmission of these two high-risk HPV in Beijing women.

In the HPV genotypes not included in the 9-valent vaccine, the HPV39 and HPV56 prevalence both changed statistically significant (*p* < 0.05). Furthermore, we performed subgroup analysis based on age. It was found that HPV16 positive rate increased in one group (the age group of 15–30), while HPV18 positive rate kept at an almost stable level in three groups (the age group of 31–45, 46–60, and >60) except for the youngest group 15–30 years. The nonsignificant decrease in HPV16 prevalence in the population group aged 46–60 years might be the beneficial effect of HPV vaccine entering the Chinese market. Some women in this age group were eligible several years ago (e.g., 2016) for vaccination and they should have a better ability to afford the vaccine fee.^11^ But we wondered why the HPV16 prevalence did not decrease in the 31–45 years age group. Women in this group were most likely to be vaccinated against HPV. This may be related to the age range of our grouping, the lifestyle of the women in this group, and their financial ability. The global burden of cervical cancer (close to 90%) has been reported to occur in developing countries.^12^ To the best of our knowledge, HPV vaccination in China was subjected to educational level, cognitive level, HPV vaccine supply, household income level, *etc*. Encouragingly, the prevalence of HPV58 appeared to decrease in the 46–60 years age group (*p* = 0.020, Table 5), although no change was shown in the general population. This could be a plausible change associated with HPV vaccination. As indicated in Table 3 to Table 6, except for the 46–60 age group, the prevalence of a few HPV types in the 15–30, 31–45, and >60 age groups has increased over the past seven years, especially in the 15–30 age group. The year 2020 was the first year that the COVID-19 epidemic began. The outbreak of COVID-19 severely affected people’s lives, including hospital visits. Therefore, we speculated that the women patients of 2020 in our study had more severe symptoms, *i.e.*, HPV infection was more severe. However, the overall prevalence of 2020 was lower than that of 2018. Although some studies indicated the importance of timely vaccination against HPV, particularly before sexual debut,^13^ we still thought that the prevalence decline in the 46–60 years group of 2020 might be the preventive effects of HPV vaccines. Gargano *et al.* evaluated HPV vaccines’ impact on cervical intraepithelial neoplasia grades decline. They found that HPV16/18-CIN2+ in 20- to 24-year-olds declined significantly in 2015–2016 compared with 2008–2009, while no significant declines were observed in older groups.^14^ This finding was partly not consistent with our results mentioned above. As far as we know, there are a large number of women in China, and the supply-demand for the 4-valent and 9-valent HPV vaccines was insufficient. The appointment cycle typically spanned a duration of 1–2 years. So the vaccination rate was presumably very low (maybe less than 20%). Furthermore, owing to the expensive cost of HPV vaccination, women around 40 years were most likely to choose the vaccination. Therefore, we speculated that the overall prevalence had indeed not yet decreased and occurred only in a certain age group of women.

To further evaluate genotype replacement and possible vaccine impact, the top five prevalence HPV types and dual-infection combination genotypes were specially analyzed, respectively. HPV52 has always been the most popular type for seven years. In other cities or provinces of China, HPV52 was most prevalent as well, such as in Sichuan, Guangdong, and Shanghai.^8^ HPV16 and 58 followed behind HPV52 as the second or the third prevalent type. The fourth type HPV39 (in 2014) prevalence gradually decreased to the eighth type in 2020 (data not shown). Whether this was a result of cross-protection by the 9-valent vaccine needs to be confirmed. Data from vaccine trials has shown that there is limited antibody cross-reactivity, except between closely related genotypes.^15^ HPV51 prevalence was the fourth position in Beijing from 2016 to 2020, which might differ from the case in other cities.^8^ HPV56 became more popular in 2018, especially among young females. This was in line with the extent of HPV56 popularity in Sichuan province. Also, HPV56 was the fifth most common genotype in Ethiopia among Ethiopian women presented with different kinds of cervical abnormalities.^16^

We noticed an evident change in HPV dual-infection combinations in our laboratory. The dual-infection of HPV39/68 was the most common dual-infection combination all the time from 2014 to 2018. However, it seemed to decrease suddenly since 2019. This might be owing to the reduction of HPV39 prevalence, although HPV68 prevalence did not reduce (Table 1). Another feature of dual infection was that the prevalence of all kinds of dual infection was very nearly the same. He *et al.* showed that the severity of abnormal cytology in an infected individual increased with the number of high-risk HPV types.^8^ Therefore, women should pay more attention to their healthy when infected with two or more HPV types.

In some regions worldwide, HPV16 and 18 were the cause of approximately 70% of all cervical cancers, as well as a subset of anogenital and oropharyngeal cancers.^17^ In Ethiopia, the combined prevalence of HPV16/18 was at 53.7% among women with different kinds of cervical abnormalities.^16^ In Italy, HPV16 (64%) and HPV18 (7%) were frequently reported from women with abnormal cervical cytology.^18^ In the city Kaifeng of China, HPV16 prevalence was at 22.5% and HPV18 prevalence was at 5.4% in women patients of gynecology and obstetrics department.^19^ But the combined prevalence of HPV16 and 18 was not very high in Beijing (Fig. 3).

## Conclusion

In conclusion, our study showed some slight variations regarding overall HPV prevalence change in different age groups and specific genotypes’ prevalence change during a long time in a big city in China. During that time, HPV vaccines were introduced to Chinese women. First, the prevalence of both HPV31 and HPV56 increased significantly and the prevalence of HPV39 decreased significantly in the overall population across the seven years. Then in the subgroups on the basis of age, the prevalence of both HPV31 and HPV56 increased significantly in the 15–30 and 31–45 age groups, the same as the prevalence changes in the overall population by year. In addition, HPV39 prevalence reduced in the 46–60 age group, which was the same as in the overall population by year, too. However, HPV58 prevalence decreased in the 46–60 age group, which was not observed in the overall population by year. Dual infection was not analyzed by age owing to few infection cases, but it was found that HPV39/68 dual infection almost disappeared in 2020. The trends need to be observed continuously.

## Data Availability

The data that support the findings of this study are available from the corresponding author upon reasonable request.

## Abbreviations Used

HPV: human papillomavirus
HSIL: high-grade squamous intraepithelial lesion
